# Child and adolescent mental health policy in South Africa: history, current policy development and implementation, and policy analysis

**DOI:** 10.1186/s13033-018-0213-3

**Published:** 2018-06-26

**Authors:** Stella Mokitimi, Marguerite Schneider, Petrus J. de Vries

**Affiliations:** 1 Division of Child and Adolescent Psychiatry, University of Cape Town, 46 Sawkins Road, Rondebosch, Cape Town, 7700 South Africa; 2 Red Cross War Memorial Children’s Hospital, Klipfontein Road, Rondebosch, 7700 South Africa; 3 Alan J Flisher Centre for Public Mental Health, Department of Psychiatry and Mental Health, University of Cape Town, 46 Sawkins Road, Rondebosch, South Africa

**Keywords:** Child, Adolescent, Mental health, Policy development, South Africa

## Abstract

**Background:**

Mental health problems represent the greatest global burden of disease among children and adolescents. There is, however, lack of policy development and implementation for child and adolescent mental health (CAMH), particularly in low- and middle-income countries (LMICs) where children and adolescents represent up to 50% of populations. South Africa, an upper-middle income country is often regarded as advanced in health and social policy-making and implementation in comparison to other LMICs. It is, however, not clear whether this is the case for CAMH. The national child and adolescent mental health policy framework of 2003 was developed to guide the establishment of CAMH policies provincially, using a primary care and intersectoral approach. This policy provided a framework for the nine South African provinces to develop policies and implementation plans, but it is not known whether this has happened. The study sought to examine the history and current state of CAMH policy development and implementation, and to perform a systematic analysis of all available CAMH service-related policies.

**Methods:**

A comprehensive search was performed to identify all provincial mental health and comprehensive general health policies across South African provinces. The Walt and Gilson policy triangle framework (1994) was used for analysis.

**Results:**

No South African province had a CAMH policy or identifiable implementation plans to support the national CAMH policy. Provincial comprehensive general health policies addressed CAMH issues only partially and were developed mainly to address the challenges with HIV/AIDS, TB, maternal and child mortality and adherence to the millennium development goals. The process of policy development was typically a consultative process with internal and external stakeholders. There was no evidence that CAMH professionals and/or users were included in the policy development process.

**Conclusions:**

In spite of South Africa’s upper-middle income status, the absence of any publically-available provincial CAMH policy documents was concerning, but in keeping with findings from other LMICs. Our results reinforce the neglect of CAMH even at policy level in spite of the burden of CAMH disorders. There is an urgent need to develop and implement CAMH policies in South Africa and other LMICs. Further research will be required to identify and explore the barriers to policy development and implementation, and to service development and scale-up in CAMH.

## Background

It is clear that mental health problems represent a substantial proportion of the global burden of disease. Child and adolescent mental health (CAMH) is slowly becoming recognized as a growing public health priority as exemplified by the WHO resolution on autism spectrum disorders in 2014 [1], recent special issues on CAMH and adolescent health in the Lancet [2] and focus on mental health in the sustainable development goals [3]. However, this recognition alone is not enough to influence policy development and implementation for CAMH services. There are other contextual factors that are influential in determining policy development and implementation, given that mental disorders represent the greatest burden of disease in children and adolescents around the world, affecting 10–20% of them [4, 5]. Furthermore, the majority of adult mental disorders develop during childhood or adolescence [6, 7] when they could potentially be prevented, or identified and treated early.

In high-income countries one in four to five young people in the general population suffer at least one mental disorder in any given year [5]. There is a relatively small evidence-base for the burden of child and adolescent mental disorders in African countries and more in low- and middle-income countries (LMICs) [5, 8]. The little evidence available shows that poverty and unemployment are risk factors for poor child and adolescent mental health and for developing CAMH disorders. Brain damage, consequent neuropsychiatric morbidity, intellectual disability and epilepsy are more common in LMICs than in high-income countries, and these disorders impact on educational attainment [13]. In South Africa various factors such as HIV infection, substance use and exposure to violence increase the risk for mental health problems in children and adolescents even further [9]. Based on data from high-income countries, the overall estimated and adjusted 12-month prevalence rates for psychiatric disorders in children and adolescents was calculated in one of the South African provinces (Western Cape) in the and estimated to be 17% in 2006 [10].

However, despite the evidence on the burden of CAMH problems, the rate of unmet needs in CAMH is still high especially in LMICs [5]. CAMH services have important roles in the prevention of mental disorders, in promotion of mental health and wellbeing of children and adolescents, in reduction of risk factors associated with mental illness, and in the provision of curative services using evidence-based strategies for those who require treatment [5, 9, 11]. Globally, the development, implementation and monitoring of CAMH services start with sound policies and planned service delivery models. Well-considered policies are required to provide a framework for service delivery relevant to particular contexts, to present appropriate and implementable systems and pathways to care, and to provide a framework for implementation, funding and on-going monitoring of such systems. Policy therefore provides a roadmap for programme development, reflects commitment from government and relevant authorities provides a mandate to support funding mechanisms, and helps to identify those accountable for service provision [12].

However, there is a lack of policy development and implementation for CAMH globally, and especially in LMICs [12]. Shatkin and Belfer summarised the state of CAMH services and policies and noted “the relatively new development of knowledge in CAMH, lack of appreciation of a developmental perspective related to CAMH disorders, stigma, fragmented advocacy constituency and reluctance of professionals to engage in debates over policy” as factors contributing to lack of policy development and implementation in CAMH [12].

### History of CAMH Policy development in South Africa

South Africa is one of the 14 out of 191 countries recognised by the United Nations, to have a clearly articulated National CAMH policy [12]. In South Africa, legislation and policy development is done at National level by the Minister of Health in consultation with a range of stakeholders. The nine provincial Departments of Health are then responsible for developing implementation plans with clear targets, indicators, budgets and timelines. Provincial departments are also responsible for monitoring and evaluation of the implemented national policy and legislation. Provincial districts (subdivisions of provinces) are responsible for the local implementation of interventions in accordance to national and provincial priorities [13].

A chronology of CAMH-related policy development and processes in South Africa is outlined in Table 1. The development of the national CAMH policy in South Africa started in 1977 with the Potgieter commission. This commission recommended intersectoral collaboration, early identification of CAMH disorders at primary health care (PHC) level and in schools, and increasing capacity of health and education staff to identify CAMH disorders [11].

**Table 1:** The chronology of child and adolescent mental health policy development and processes in South Africa

Year	Policy development
1977 (Apartheid era)	The Potgieter commission emphasized intersectoral collaboration, early identification of CAMH disorders at primary healthcare level and in schools, and increasing capacity of teachers and health staff to identify CAMH disorders [14]
1994 (post-apartheid era)	The first democratic President acknowledged the importance of children and their vulnerability [14]
1997	White paper for the transformation of the health system in South Africa [15]
	National Policy guidelines for improved mental health care [16]
2001–2003	National policy guidelines for youth health and CAMH [14]
2003	National CAMH policy guidelines, 2003 [14]
2002	National mental health care act, no. 17 of 2002 [17]
2003	Norms and standards to develop CAMH services [18]
2005	A situational analysis of CAMH services in South Africa [19]
2008	The draft of the strategic mental health plan for the Northern Cape Province finalised by the national multidisciplinary committee (NHC), awaiting adoption as of 2016 [20]
2012	The national mental health summit facilitated the adoption of the national mental health policy guidelines to improve mental health care [13]
2012	The mental health summit adopted the “Ekurhuleni declaration” [13]
2013	The “mental health policy framework and strategic plan” was formally adopted for implementation [13]

**Fig. 1:**
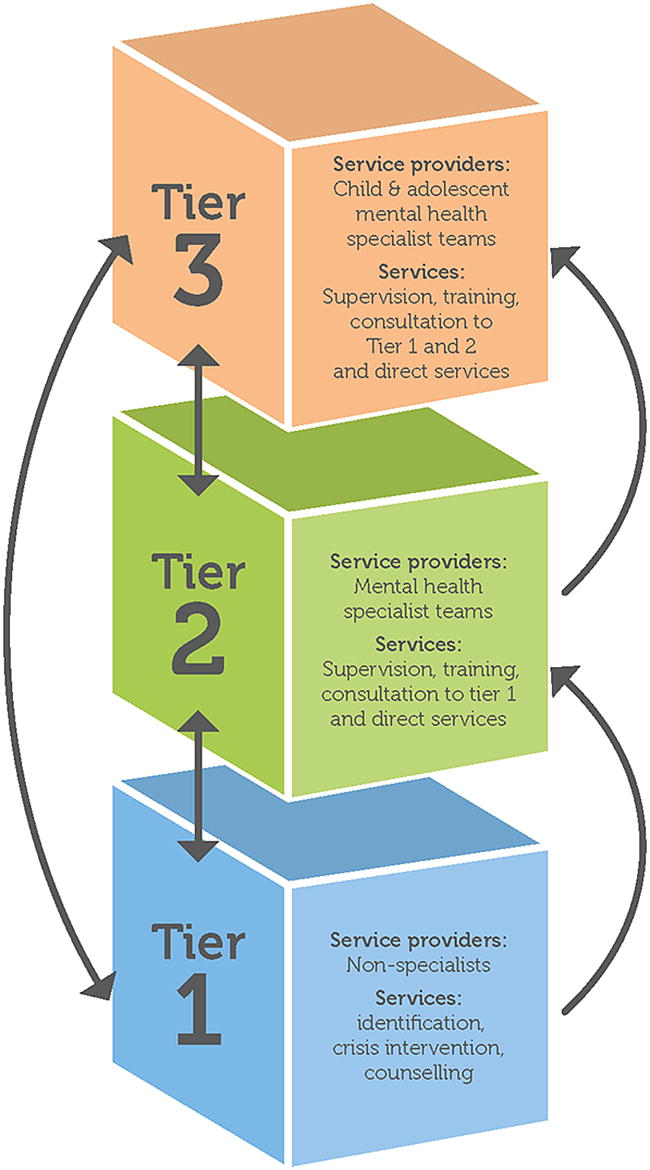
The three-tier model for CAMH services in South Africa

The policy guidelines for youth health and CAMH were developed between 2001 and 2003, after the development of guidelines for planning child and adolescent policy in developing countries by Desjarlias and colleagues [11]. The national CAMH policy framework of 2003 [14] was developed to guide the establishment of CAMH policies provincially, using a primary care and intersectoral approach. The policy set out a three-tier model for CAMH services and outlined the movement of children between these tiers. The first point of contact for patients should be at level 1 (informal and formal primary health services), and then move to level 2 or 3 depending on the complexity of the problem. Patients will move between these levels based on the complexity of the problem, the type of assessment, and/or the type of intervention needed. Provincial implementation plans to support this policy were recommended as the next step [19]. See Fig. 1.

The last published situational analysis conducted by Kleintjes and colleagues in 2005 [19] in four African countries (Uganda, Zambia, Ghana and South Africa) assessed the CAMH resources and the issues impacting on policy, legislation and service development and implementation for CAMH. This situational analysis showed that at a national level, South Africa had mental health policy guidelines [16], a stand-alone CAMH policy [14], and mental health legislation [17]. However, the national mental health policy did not provide specifically for children and adolescents, and the legislation did not include CAMH issues. The legislation addressed only one out of six provisions recommended for the protection of minors by the WHO legislation checklist [21] and while it recommended age-appropriate services, there were no implementation plans to support the CAMH policy [19]. Kleintjes and colleagues concluded that this situation was due to lack of adoption of the overarching national policy guidelines for improved general mental health care [19].

The national policy guidelines for improved general mental health care of 1997 were formally adopted in July 2013, and led to the development of the national mental health policy framework and strategic plan 2013–2020. The national mental health committee planned to support all the provinces to develop their own mental health plans [20].

Kleintjes and colleagues in 2005 also found that at a provincial level, only one of the nine South African provinces (Northern Cape) had a draft mental health implementation plan. None of the provinces had implementation plans to support the CAMH policy but many were using the National legislation to guide service provision. This situation was also due to lack of capacity at provincial level [19]. The draft was under construction at the time of the work by Kleintjes and colleagues in 2005. The final draft of the Northern cape strategic mental health plan was completed in 2008, and was still awaiting formal adoption as of February 2017 [22].

A recent review paper [23] investigated potential barriers to the implementation of the national mental health policy [13]. These barriers include concerns about the feasibility and sustainability of policies, other activities and policies required to ensure full integration of mental health into the health system [23], lack of financial and human resources, the limited number of evidence-based psycho-social treatment protocols for disorders such as depression and anxiety, limited awareness of and negative attitudes towards mental disorders, and the low level of health-system readiness to integrate mental health care [23].

To develop and implement CAMH policies, the mental health and poverty project (MhAPP) study recommended government commitment, capacity-building of all relevant service providers, service users, and researchers, to lobby for implementation of CAMH policies and plans, multisectoral collaboration, and raising awareness of mental health. Whilst there has been some progress at a national level and some provincial activity occurred, the development and implementation of CAMH policy was noted to be lacking in 2010 [19].

The purpose of this study was two-fold: firstly, to determine whether South African provinces have developed provincial CAMH policies and implementation plans based on the national CAMH Policy; secondly, to perform a policy analysis of all identified CAMH-related policy documents.

## Methods

### Search strategy

In order to identify all publically-available policy documents related to CAMH two search strategies were used. Firstly, web-based searches were performed of the national and all provincial departments of health websites. Searches were conducted in June 2016 and September 2016. All potentially relevant information was downloaded for analysis. Search terms included “child”, “adolescent”, “mental health”, “policy development”, “policy implementation”, “integrated school health services”, “intellectual disability”, “CAMH policy”, and “health policy”. We searched for the latest version of the provincial stand-alone mental health and CAMH policies, and for broad, inclusive or comprehensive general health documents.

In parallel with the web-based search, a stakeholder-based search strategy was used. Key staff at the national department of health, academics involved in mental health policy, and senior clinicians in CAMH were contacted to obtain the names and contact details of responsible individuals and policy stakeholders at national or provincial level. All identified policy stakeholders were contacted telephonically and by email by the first author (SM) to obtain the most recent policy-related documents.

### Data extraction and analysis

**Fig. 2:**
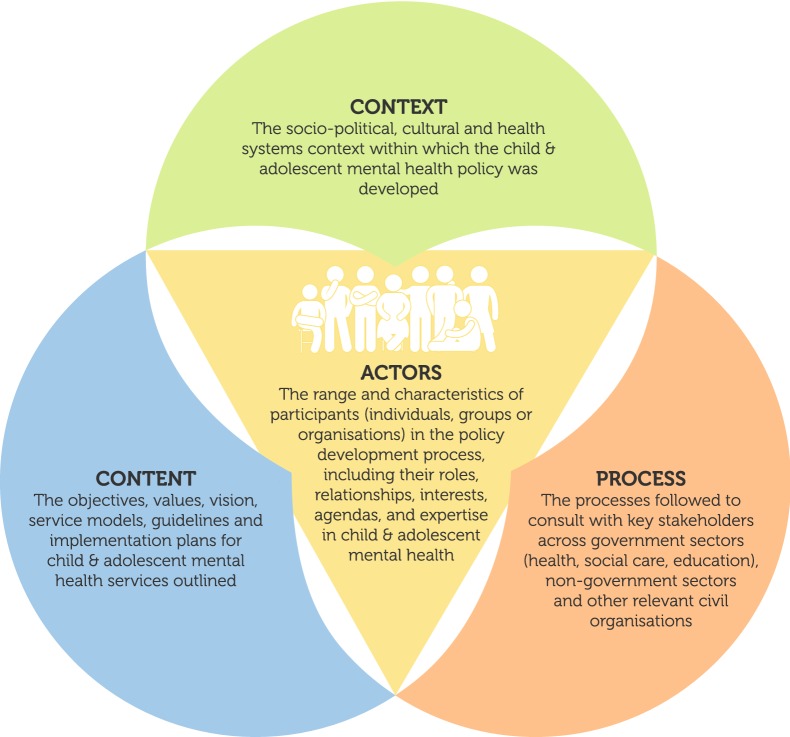
The Walt & Gilson (1994) policy triangle model

The Walt and Gilson “policy triangle model” [24] was used as framework for extraction and analysis of identified policy documents. Walt and Gilson’s “triangle model” is a useful model for analysing a variety of health issues including mental health issues. It focuses on the content of policy, range of actors, context and processes and the interaction between these elements in policy making and policy implementation. The model provides a framework for understanding the process of health policy reform and to plan for effective implementation [24]. The model can be used retrospectively and prospectively. Figure 2 shows the policy triangle model as adapted from Walt and Gilson [24].

All obtained provincial documents were read, and data extracted focusing on the content, the context, the process of policy development as well as the actors involved in developing the policy. No formal interviews were conducted to obtain further information, beyond the contacts made to access the documents.

## Results

### Policy documents identified

**Fig. 3:**
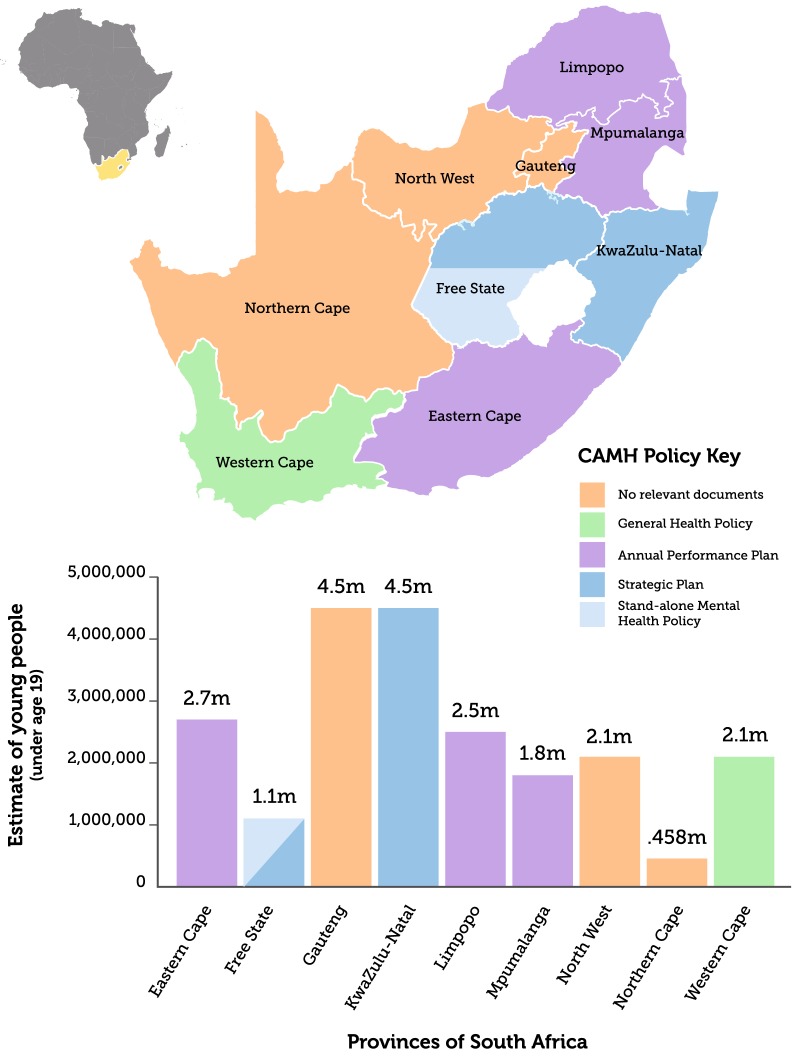
Geographical distribution of identified CAMH-related documents, and the number of children and adolescents across the nine South African provinces [31]

Table 2 provides a short definition of the types of policy-related documents identified and Table 3 lists all documents identified. Figure 3 shows the geographical distribution of the identified policy-related documents across the nine South African provinces and indicates the number of children and adolescents (< 19 years) per province.

**Table 2:** Short definitions of the different types of policy-related documents identified

Policy document	Explanation of the document
Stand-alone mental health policy	Defines the vision for the future mental health of the population, specifying the framework which will be put in place to manage and prevent priority mental and neurological disorders
Stand-alone child and adolescent mental health policy	Defines the vision for the future mental health of the children and adolescents, specifying the framework which will be put in place to manage and prevent priority mental and neurological disorders
CAMH plans	Is a pre-formulated detailed scheme to implement the vision and objectives defined in the child and adolescent mental health policy. It includes the concrete strategies and activities to be implemented and specifies targets to be achieved by the government. It clarifies the roles of the different stakeholders in implementing the activities of the mental health plan
Mental health legislation	Regulates mental health care co-ordinates access to services. It sets out the rights and duties of patients and providers, and explains how the property of mentally ill persons may be dealt with in a court of law
General health policy	Defines the vision for the future health of the population, specifying the framework which will be put in place to manage and prevent priority health disorders
Strategic plan	Outlines the broad strategic goals for the department
Annual performance plans	Sets out a framework to align strategic plans and annual performance plans. Puts emphasis on the outcomes oriented monitoring and evaluation approach

**Table 3:** The national and provincial policy documents identified from the nine South African provincial department of health websites

Policy document	National level	Provincial level								
	South Africa	Western Cape	Eastern Cape	Kwa Zulu Natal	Northern Cape	Limpopo	Mpumalanga	Free State	North West	Gauteng
Stand-alone mental health policy	Mental health policy framework and strategic plan 2013–2020 [13]	X	X	X	X	X	X	Provincial mental healthcare (22 January 2001) policy. 2004 year of review: 2009 #outdated	X	X
Stand-alone child and adolescent mental health policy	Child and adolescent mental health policy guidelines 2003 [14]	X	X	X	X	X	X	X	X	X
CAMH plans	None	X	X	X	X	X	X	X	X	X
Mental health legislation	National mental health act no. 17 of 2002 [17]									
Other comprehensive general health care documents										
General health policy		Healthcare 2030: the Road to Wellness [25]	X	X	X	X	X	X	X	X
Strategic plan			X	Strategic plan 2015–2019 [26]	X	X	X	Free state strategic 5 year plan 2010/11–14/15 [27] #outdated	X	X
Annual performance plans			Annual performance plan 2013/14–15/16 [28]	X	X	Annual performance plan 2008/09–2011(March 2008) [29] #outdated	Annual performance plan 2016/17 [30]		X	X

X denotes that the provincial document could not be found. # denotes an outdate document

#### Mental health policy

At national level, a national mental health policy [13] exists and children and adolescents are implicitly included in this policy. The Free State province had an outdated stand-alone mental health Policy [32] which was due for review in 2009. The updated version could not be accessed from the provincial website. The other eight provinces had no mental health policies.

#### CAMH policy

At national level, a national CAMH policy [14] was still in place. We were not able to identify a stand-alone provincial CAMH policy in any of the nine South African provinces, and there was no evidence of efforts to integrate the national CAMH policy into provincial general health policies.

#### Implementation plans

None of the nine provinces had implementation plans to support the national CAMH policy. Two provinces (Western Cape and KwaZulu-Natal) acknowledged in their general health policies and plan the need to separate children and adolescents from adults, and to strengthen CAMH capacity within the general service platforms. The other seven provinces had no documented implementation plans to support the National CAMH policy.

#### Strategic plans and annual performance plans

Only the Mpumalanga province annual performance plan 2016/17 showed evidence of proactive strategies to promote mental health and increase the number of patients screened for mental disorders and increasing the number of mental health teams. There was no specific mention of CAMH in the Mpumalanga or any other provincial strategic/annual performance plans.

### Policy analysis using the Walt and Gilson policy triangle

#### The content

The content of all identified policy-related documents are summarised in Table 4. The outdated Free State mental health policy mainly focused on general mental health and did not make specific reference to children and adolescents. All the nine provinces mainly focused on general health and strengthening Primary Health Care services using intersectoral collaboration, focusing mainly on HIV and AIDS, TB and maternal and child health. There were no specific references to children and adolescents with mental health problems, and no clear guidelines for service provision for CAMH. The needs of children and adolescents with mental health problems were provided for within the general health population. Only the Western Cape, KwaZulu-Natal and Eastern Cape made specific reference to child and adolescent mental health disorders and the need to develop service for them.

**Table 4:** Content analysis of the provincial mental health and general health policy documents

Mental health and comprehensive general health document	Free State		Western Cape	Limpopo	KwaZulu-Natal	Mpumalanga	Eastern Cape
	Mental health care policy and procedures policy. 2004. Due for review: 2009 #outdated	5-year strategic plan 2010/11–2014/15 #outdated	Healthcare 2030: the Road to Wellness	Annual performance plan 2008/09–2011 #outdated	Strategic plan 2015–2019	Annual performance plan 2016/17	Annual performance plan 2013/14–2015/16
Year	2009	2010	2014	2008	2015	2016	2013
Content focus	Emphasis is on provision of mental health services at all levels of care using intersectoral collaboration between correctional services, justice, social development, education, non-profit organisations, and groups of beneficiaries	Seven strategic goals 1. Provision of strategic leadership and creation of social compact for better health outcomes 2. Improve the quality of health care services 3. Reduce the burden of disease 4. Revitalisation of physical infrastructure 5. Improve human resource management 6. Overhaul the health care system and improve its management 7. Research and development	Focuses on strengthening primary health care (PHC) and district hospitals service as well as integration of services CAMH falls into general health plans The document focuses on reducing HIV and TB, improving healthy lifestyle, preventing injuries and violence, improving maternal and child health, strengthening child health, and improving mental health	Policies and programmes are mainly focused on primary health care, district health care, hospitals and resource management. The main focus is on reducing morbidity and mortality arising from communicable diseases, vaccination of preventable childhood diseases, diseases of lifestyle, HIV and AIDS and TB, trauma, and violence against women and children	Provision of sustainable, coordinated and integrated comprehensive health system at all levels using the primary healthcare approach through the district health system	Reference is made to promoting mental health and increasing the number of patients screened for mental disorders and increasing the number of mental health teams	The focus is on re-engineering of the PHC and strengthening of emergency medical services, pharmaceutical and hospital services
Service provision plans and clear guidelines for CAMH	No specific reference to children and adolescents	Some mention of MH services and plans	No. An acknowledgement of the need to separate children with mental health disorders from adults and to develop service for them in future	No	Acknowledges CAMH disorders but unclear service provisions	No	Some recognition of the CAMH disorders but no clear plans for service provision

Northern Cape, Gauteng and North West provinces were excluded as no relevant documents were identified

#### The context

The context within which these general health policies were developed are summarised in Table 5. The contexts varied, but were mainly based on the need to mitigate the challenges with HIV and AIDS, TB and maternal and child mortality, the demand for quality general health services, and the need to adhere to the Millennium Development Goals. No reference was made to child and adolescent mental health.

**Table 5:** Context analysis of the provincial mental health and general health policy documents

Province	Free State		Western Cape	Limpopo	KwaZulu-Natal	Mpumalanga	Eastern Cape
Policy document	Mental health care policy and procedures policy. 2004 year of review: 2009 #outdated	5-year strategic plan 2010/11–2014/15 #outdated	Health care 2030	Annual performance plan 2008/09–2011 #outdated	Strategic plan 2015–2019	Annual performance plan 2016/17	annual performance plan 2013/14–2015/16
Year	2009	2010	2014	2008	2015	2016	2013
Context	The mental health care act, 2002 No. 17 of 2002) framework part of the legislative mandate	The need to address service delivery challenges comprehensively	The policy is driven by the following Changes in the external environment (demography, socio-economic determinants of health, burden of diseases and its associated risk factors such as climate change, advances in technology and limited resources) Changing policy environment and policy imperatives such as millennium development goals (MDGs), the 2030 national development plan (NDP), the priority national health outcomes and the provincial strategic objectives to improve wellness Need to ensure continuous improvement in the patient experience and providing quality health services as well as caring for the staff	The plan is based on the 5-year strategic plan aligned to the departmental service transformation plan that provides long-term vision for the provision of health services in the province	The national development plan 2030, the medium strategic framework 2014–2019, the provincial growth development plan 2030, the 2015 cabinet Lekgatlo resolutions, other sector priorities and the burden of diseases and demand for service shaped the document	To put systems in place to ensure effective service delivery	Taking responsibility to support the country effort in the realization of millennium development goals (MDGs), to mitigate HIV and AIDS and TB, as well as challenges around maternal and child mortality

#### The process and actors

All nine provinces engaged in a consultative process with various internal and external stakeholders (including non-governmental organisations and private sector) prior to endorsement by the Provincial Cabinet (see Tables 6 and 7). Various approaches were used, such as responding to stakeholder needs from the ‘bottom up’, responding to national priorities in a ‘top-down’ approach, and through comprehensive reviews of previous policies, situational analyses, and weighing up of different alternative policies. However, we were not able to find documented evidence that any child and adolescent mental health experts, service users (parents or children) or CAMH-related non-profit organisations were consulted or included in the process.

**Table 6:** Process analysis of provincial policy development

Province	Free State		Western Cape	Limpopo	KwaZulu-Natal	Mpumalanga	Eastern Cape
Policy document	Mental health care policy and procedures policy. 2004 year of review: 2009 #outdated	5-year strategic plan 2010/11–2014/15 #outdated	Health care 2030	Annual performance plan 2008/09–2011 #outdated	Strategic plan 2015–2019	Annual performance plan 2016/17	Annual performance plan 2013/14–2015/16
Year	2009	2010	2014	2008	2015	2016	2013
Process	Various options were weighed. i.e. vertical program and a decision was taken to choose an option that will embrace the primary health care approach, and bring services closer to the people within the available resources The Free State community psychiatric approach was revised to be in line with the primary health care approach	Extensive consultation within and between clusters (workshops and task teams) with top management structures, information systems and service delivery components. The document is approved by the acting HOD and the MEC	The department’s preliminary thinking was shared in a draft document circulated for public comment in 2012 and again in December of 2013 Facilitated dialogue sessions were convened with a range of external stakeholders and staff through sessions by the geographic service area management structures Many submissions were received on both occasions and colleagues raised interesting, relevant and creative ideas during the dialogue sessions All comments were considered and the written comments were individually responded to. It was endorsed by the provincial cabinet	No relevant data	The strategic plan was formulated through an extensive consultative process with internal and external stakeholders and was endorsed by the provincial cabinet The process of formulating the strategic plan was done in four phases Phase 1. performance reviews (April–July 2014) Phase 2. strategic vision and strategic priorities 2015–2019 (August–September 2014) Phase 3: top-down-Bottom-up consultation to refine provincial Priorities (October–November 2014) Phase 4: finalising and tabling the document (December–February 2015)	No consultation with external stakeholders	Consultation with various stakeholders

Northern Cape, Gauteng and North West provinces were excluded as no relevant documents were identified

**Table 7:** Actors involved in development of the provincial mental health and general health policy documents

Province	Free State		Western Cape	Limpopo	KwaZulu-Natal	Mpumalanga	Eastern Cape
Policy document	Mental health care policy and procedures policy. 2004 year of review: 2009 #outdated	5-year strategic plan 2010/11–2014/15 #outdated	Healthcare 2030: the Road to Wellness	Annual performance plan 2008/09-2011 #outdated	Strategic plan 2015–2019	Annual performance plan 2016/17	Annual performance plan 2013/14–2015/16
Year	2009	2010	2014	2008	2015	2016	2013
Actors	Assistant manager (mental health and substance abuse); manager (personal health); and various unspecified stakeholders from all other departments and at all levels from bottom up	Top management structures, management structures, acting HOD and MEC	Unspecified internal and external stakeholders (the public, geographic service area management teams, provincial cabinet	The works of the department were coordinated by the head office which provides the legislative interface between the governments, civil society and other relevant unspecified stakeholders, and provides strategic direction and overall management and administration of the department	The unspecified internal and external stakeholders	The document was developed by the provincial department of health in Mpumalanga, under the guidance of the MEC	Department of health
Inclusion of child and adolescent mental health expects and users in the formulation of these policies	No data	No data	No data	No data	No data	No data	

Northern Cape, Gauteng and North West provinces were excluded as no relevant documents were identified

### Comparison of findings to previous analysis in 2010

Table 8 shows a comparison of the previous situational analysis [19] and the current state of CAMH policy development and implementation at provincial level. The results showed that there is still no provincial mental health or child and adolescent mental health policies in any of the nine provinces. The national legislation is still used to guide service provision. The Northern Cape provincial mental health policy is still awaiting formal adoption.

**Table 8:** A comparison between the 2010 situational analysis and the current state of CAMH policy development and implementation at provincial level

Documents	Kleintjes et al. [19]		Current state of CAMH policy development and implementation (this study)	
	Nationally	Provincially	Nationally	Provincially
Mental health policy	National mental health policy guidelines of 1997 (not formally adopted)	None	National mental health policy guidelines of 1997 formally adopted in 2013	None
Mental health plans	None	Northern cape mental health draft plan	“Mental health policy framework and strategic plan 2013–2020”	None Northern Cape mental health plan finalised 2008 but not yet officially adopted
Child and adolescent mental health policy	Child and adolescent mental health policy guideline 2002	None	Child and adolescent mental health policy guideline 2002	None
Child and adolescent mental health plan	None	None	None	None
Mental health legislation	Mental health act no. 17 of 2002	National mental health act no. 17 of 2002	Mental health act no. 17 of 2002	National mental health act no. 17 of 2002
Provision for the protection of minors in national legislation	The legislation addressed only one out of six provisions recommended for the protection of minors by the WHO legislation checklist	The legislation addressed only one out of six provisions recommended for the protection of minors by the WHO legislation checklist	The legislation addressed only one out of six provisions recommended for the protection of minors by the WHO legislation checklist	The legislation addressed only one out of six provisions recommended for the protection of minors by the WHO legislation checklist
Inclusion of child and adolescent mental issues in national legislation	No	No	No	No

## Discussion

The aim of this paper was to examine the current state of CAMH policy development and implementation in the nine provinces of South Africa, and to perform a policy analysis of all CAMH-related policy documents. We started with a brief history of events that led up to the development of a National CAMH policy. We expected that, after the formal adoption of the overarching National CAMH policy, all provinces would have clear CAMH policies and implementation plans to support the National CAMH policy, but this was not the case. None of the nine provinces had a current CAMH policy or plan. Only the Western Cape and KwaZulu-Natal provinces overtly acknowledged the need for plans to separate children from adults and to attend to the specific needs of children and adolescents with mental health disorders.

Using the Walt and Gilson policy analysis triangle (1994), we examined the content, context, processes and actors involved. In terms of content analysis, none of the nine provinces addressed the specific needs of children and adolescents with mental health problems. Where CAMH was mentioned, it was very superficial and non-specific. There were no clear guidelines and plans for service provision. With regard to the context under which these provincial policies were developed, drivers were predominantly the burden of HIV and AIDS, TB and maternal and child mortality, the demand for quality general health services, and the need to adhere to the Millennium Development Goals. Regarding processes involved in policy development, we observed a range of approaches (bottom up-and top-down) used by provinces to engage with often unspecified internal and external stakeholders and with non-governmental organisations to develop policies. However, from the document review, we were not able to identify any clear evidence that any CAMH experts and/or CAMH users (parents or young people) were included as actors in the process.

While there has been progress at national level since the last study by Kleintjes and colleagues with regards to the formal adoption of the overarching national mental health policy, findings at provincial level were essentially unchanged since the last study in 2005 [19] and shows clear evidence of on-going neglect of CAMH policy development and implementation at provincial level. Examples such as the lack of adoption of the draft mental health plan in the Northern Cape since finalization in 2008, and lack of explicit inclusion of CAMH in the provincial general health policies raise major concerns about content and implementation of mental health policies in South Africa, and, in particular, with regards to the mental health of children and adolescents.

We acknowledge that there may be many barriers to policy development and implementation in LMICs. Some of the barriers to the implementation of the National policy identified by Schneider and colleagues [23] included lack of capacity of staff, shortage of staff, inadequate finance, and the burden of mental disorders, and child and adolescent mental disorders.

However, the lack of policy development and implementation in CAMH may exacerbate CAMH problems [12] and impact negatively on service delivery. Nearly forty percent (40%) of all South Africans are under the age of 18 years and the mental health burden is of great concern for this sector of the population. There is therefore an urgent need for action to recognise CAMH services as a health priority, and for the South African government to mandate the development of appropriate and relevant CAMH policies, implementation and monitoring plans.

These findings highlight an urgent need for each province to develop CAMH policy and implementation plans to give effect to the National CAMH policy. While we acknowledge the barriers to CAMH policy development and implementation [12–14, 23, 33], we advocate that CAMH policy and implementation plans are still required to provide a framework for service delivery which will be relevant to the needs of young people.

One way to do this is for the provincial government to commit to incorporating research findings into planning and policy development. This requires a close relationship and engagement between the provincial government and the researchers. Research on the current state of CAMH service is required in each province. The first step is to conduct a situational analysis of CAMH services at provincial level in order to map the current state of CAMH services, to identify the gaps and the need. Secondly, the stakeholders in CAMH services i.e. users and providers should be engaged in order to gather their lived experiences and perceptions of the CAMH services that are offered to them, and to contribute to the recommendations for policy development. Lastly, researchers should engage policy makers with the findings in order to ensure policy planning and implementation.

## Conclusions

In spite of the upper-middle income status of South Africa, the absence of any provincial CAMH policy and plans were deeply concerning, but, sadly in keeping with findings from other LMICs. Findings reinforce the widespread neglect of CAMH even at policy level, in spite of the well-recognized burden of CAMH disorders.

We acknowledge that we were only able to analyze documents that were publicly available. Documents not officially adopted and those not publicly available were not included in the analysis. It is therefore possible that there may have been relevant documents that are up to date that we could not access. However, we would argue that provincial and national policies should be readily and electronically available in the spirit of transparency and to facilitate communication and implementation of policies.

There is an urgent need for development and implementation of provincial CAMH policies and implementation plans in South Africa and LMICs. Further research will also be required to identify and explore the barriers that continue to prevent CAMH policy and service development, and scale-up.

## Abbreviations

CA: children and adolescents
CAMH: child and adolescent mental health
DoH: department of health
MH: mental health
NHC: national health committee
LMICs: low and middle income countries
